# Characterizing and assessing compliance of online vendors to the state of Massachusetts ENDS product sales ban

**DOI:** 10.18332/tid/131199

**Published:** 2021-01-20

**Authors:** Matthew C. Nali, Vidya Purushothaman, Qing Xu, Raphael E. Cuomo, Timothy K. Mackey

**Affiliations:** 1 Global Health Policy Institute, San Diego, United States; 2 Department of Anesthesiology, University of California San Diego School of Medicine, San Diego, United States; 3 Master’s Program in Public Health, University of California San Diego School of Medicine, San Diego, United States; 4 Department of Healthcare Research and Policy, University of California San Diego - Extension, San Diego, United States; 5 Division of Infectious Diseases and Global Public Health, Department of Medicine, University of California San Diego School of Medicine, San Diego, United States

**Keywords:** Electronic Nicotine Delivery System, vaping, online vaping shops, Massachusetts ban, electronic cigarette

## Abstract

**INTRODUCTION:**

Recent reports of lung injury associated with Electronic Nicotine Delivery System (ENDS) products precipitated by increasing vaping prevalence and interest in flavors among adolescents has led to policies that restrict the sale, distribution, and accessibility of ENDS products. This study assessed compliance of online ENDS vendors to the Massachusetts temporary sales ban.

**METHODS:**

The study involved structured web surveillance for online ENDS vendors using keyword searches on Google search engine (October to November 2019.) Once vendors were identified, we conducted simulated online purchases, defined as placing an order for an ENDS product by putting it in the website shopping cart without finalizing payment. Simulated purchases and content analysis of websites was conducted to determine compliance characteristics. Fisher’s exact test was used to identify associations between compliance and website characteristics such as location and age verification requirements.

**RESULTS:**

Simulated online purchases from 50 identified ENDS vendors yielded 72% (n=36) stores that were non-compliant and allowed placement of ENDS product orders, without restrictions, to a Massachusetts address. The remaining 14 websites had processes in place to prevent orders from buyers located in Massachusetts. Other characteristics of interest, including use of age verification, location data, and web registrar/registrant data were collected and reported.

**CONCLUSIONS:**

The September 2019 Massachusetts executive order was a comprehensive ban on selling ENDS products both online and offline. However, our study found that close to three-fourths of the vendors appeared to be non-compliant, indicating that implementation and enforcement are ongoing challenges for future tobacco control efforts on the internet. Policymaking needs to be specifically tailored to address the unique challenges of online environments, particularly in the context of identifying non-compliant sites, ensuring age verification, and addressing non-US sellers.

## INTRODUCTION

In September 2019, the Massachusetts governor declared a public health emergency and ordered a 4-month ban on the sale of all ENDS products within the state (the 15th most populated state in the US). This decision was in direct response to growing concerns associated with the nationwide outbreak of e-cigarette or vaping product use-associated lung injury (EVALI)^1^. However, it was not the first or only state to try to ban specific ENDS products for public safety concerns, which now include 10 states and over 300 localities^2^.

Vaping prevalence has significantly increased over the last few years among youth and young adults compared to older age groups^3,4^. Critically, the EVALI outbreak has been characterized as impacting young adults disproportionately, with the majority of cases reported among this critical demographic (median age of cases was 24 years old and 80% of the cases were aged <35 years) as well as cases among adolescents (15% aged <18 years), both being high-risk groups for tobacco initiation^5–7^. Additionally, the U.S. Centers for Disease Control and Prevention (CDC) reports that 82% of the 2022 hospitalized EVALI patients had used tetrahydrocannabinol (THC)-containing products, evidencing additional patient safety risk associated with this psychoactive compound^8^.

Prior to and following the EVALI outbreak, local, state and federal regulatory bodies have attempted to limit the potential harm of ENDS products (including hundreds of reports of e-cigarette burns and nicotine poisoning), particularly among youth and adolescents, by restricting the sale, marketing and access to various ENDS devices, supplies and products^1,9^. In mid December 2019 following a peak of EVALI cases, the U.S. President signed into law legislation that raised the minimum age requirements for tobacco or tobacco-related product purchases from 18 to 21 years of age and also issued federal rules restricting major retailers from selling all fruit and sweet-flavored ENDS after backtracking on plans for a more comprehensive flavored sales ban^10^. The U.S. Food and Drug Administration (FDA) has also acted, issuing policy guidance in January 2020 about its enforcement action against the manufacture, distribution, and sale of unauthorized flavored cartridge-based e-cigarettes^11^. Beyond the federal government, various state-level policies were also implemented in response to growing youth vaping popularity and rise of EVALI cases, such as a prohibition on the sale of flavored e-cigarettes in the state of New York and similar policies in different local municipal communities in California^12-15^. Some of these policy responses address only physical access (e.g. brick-and-mortar and ENDS storefronts), while others also attempt to address digital sales channels (e.g. internet, e-commerce platforms, social media, etc.)

Through the governor’s executive order, the state of Massachusetts implemented a complete emergency sales ban, both in-store and online, meaning it was the only US state expressly prohibiting the sale of all ENDS products, excluding medically prescribed marijuana products. News about the ban was disseminated primarily via press release through the governor’s office and the Department of Public Health. It was proposed to last for 4 months (24 September 2019 to 25 January 2020) with an option to extend the ban if needed but ended early on 11 December when new Massachusetts legislation replaced the executive order and instituted a restriction on only flavored ENDS product sales. The executive order included three primary provisions: 1) a seller located in Massachusetts may not make an in-store sale of ENDS products to a consumer located in Massachusetts; 2) a seller located in Massachusetts or a seller located in any other State may not make a sale of ENDS products by online, phone, or other means for delivery to a consumer located in Massachusetts; and 3) a seller located in Massachusetts may make a sale of ENDS products by online, phone or other means for delivery to a consumer located in another State^1^.

Based on the stipulations of the executive order, any seller of ENDS products in the US was prohibited from selling online to a consumer located in Massachusetts. In contrast, sellers located in Massachusetts were allowed to sell online to consumers in other States. Hence, the sales ban was limited to consumers living in Massachusetts, essentially residents within the state’s legal purview. It is not clear from the executive order if sellers located in other countries are explicitly prohibited from selling to Massachusetts residents, though importation of ENDS products from outside the US is subject to separate regulation and enforcement by federal agencies, such as the U.S. Food and Drug Administration, the Department of Homeland Security, and Customs and Border Protection. Importantly, international online tobacco and ENDS vendors may fail to adhere to federal and state legal requirements (though importation of tobacco products is subject to FDA oversight), including age restrictions, marketing and promotion regulations, and enabling access to cheaper products^16^.

The temporary Massachusetts executive order provides a unique opportunity to assess compliance and specific vendors characteristics to a comprehensive sales ban in the online ENDS selling environment. Previous studies have conducted content analysis of online ENDS and tobacco vendors to identify vendor characteristics (e.g. location, types of online stores, payment processes, shipping options), product offerings and pricing, marketing strategies, age verification processes, use of social media, and health claims made but not in the context of a comprehensive sales ban that covers all ENDS products^17-23^. Building on these prior studies this study conducted structured web surveillance and simulated purchases to specifically characterize compliance of online ENDS vendors to the state sales ban and identify characteristics of domestic and international vendors by using IP addresses and other website information. The purpose of this study is to generate preliminary evidence of potential challenges associated with implementation of state ENDS sales ban policies.

## METHODS

The study was conducted in two phases: 1) internet-based surveillance and content analysis to identify and characterize online ENDS vendors; and 2) simulated shopping to test the ability of identified online ENDS vendor websites to receive online orders to Massachusetts consumer addresses, which at the time had in place an ENDS sale ban issued by executive order. Internet surveillance was conducted from 29 September to 7 November 2019 and simulated purchases were conducted on 1 October, 9 October and 7 November 2019. Ethics approval was not applicable/not required for this study. All information collected from this study was from the public domain and the study did not involve any interaction with users. Any user indefinable information was removed from the study results. Data collected on internet platforms are available upon request from the authors, subject to appropriate de-identification.

### Internet surveillance and website content analysis

The first phase of the study involved conducting structured online search queries using the incognito feature on the Google Chrome browser and Google search engine to identify websites that directly sold ENDS products to consumers. Incognito mode was used from a US-based Internet Protocol (IP) address with user cookies deactivated and search history turned off to prevent the influence of any prior search history on the browser and to simulate a typical user online search for online sales of ENDS products. The keywords used to conduct searches were based on popular keyword search queries related to e-cigarettes, as analyzed in Google Trends, as well as keywords discovered by browser recommendations for the phrase ‘buy vaping products online’. Organic search results (website description and hyperlinks) for the first six pages of each search query string were then analyzed to identify online ENDS vendors and also select website characteristics. A previous study conducted in 2015 that examined the characteristics of online ENDS vendors was adopted for the structured internet search query and content analysis approach in this study^24^.

Content analysis of hyperlinks and websites was conducted to first identify online vendors actively selling ENDS products direct-to-consumer. Websites were assessed to determine if they were actively selling ENDS products, what age verification process was used, and physical storefront address listed. Age verification was based on three categories including: 1) a website with no age verification method; 2) a verification method that cannot effectively verify age due to lack of sufficient data disclosure or collection (i.e. a simple click through that someone is above the legal age); and 3) a form of online age verification service. Specifically, websites were only included if they offered the sale of ENDS products and included an e-commerce shopping cart to effectuate payment and purchase. For all vendors identified, The Internet Corporation for Assigned Names and Numbers (ICANN), a nonprofit organization responsible for coordinating the maintenance and administration of the name space of the internet WHOIS look-up tool (a directory of domain registrant information) was queried to obtain the website domain name, registrar name, registrant name, registrant county, registrant address, IP organization, IP server, and creation date for websites.

### Simulated purchases

After identifying and characterizing online ENDS vendors, the first author conducted simulated online purchases (which terminated upon request for confirmation of payment) to assess whether it was possible to advance through the online ordering process for the purposes of buying and shipping an ENDS product to a Massachusetts consumer address. Simulation of ENDS product orders was conducted by selecting ENDS starter kit (including a mod, tank, coils and replacement parts) and placing it in the shopping cart of these websites. ENDS starter kits were chosen on the basis of widespread product availability on all websites identified compared to more limited availability of specific ENDS products. Procedure for simulated shopping included selecting and placing the starter kit in the website shopping cart, advancing through the account registration process, entering shipping information for an address in Massachusetts, and generally confirming ordering information without issuing payment. Additionally, each online ENDS vendor website was searched to identify if any THC-containing ENDS products were available to purchase and could be placed in the shopping cart. We included THC products as they represent a potential and elevated patient safety risk due to their strong association with EVALI cases.

Online ENDS vendors that allowed shipping to a Massachusetts address were categorized as non-compliant and those that had controls to prevent sale/shipment to Massachusetts were categorized as compliant. Fisher’s exact test was used to identify any possible associations between online ENDS store sales ban compliance and website characteristics to determine if there were any significant proportional differences between compliant and non-compliant online ENDS vendors. The website characteristics tested included IP server location, age verification requirement, hosting company type, and shipment restrictions. These characteristics were chosen on the basis of assessing whether non-US websites, those that did not use age verification (another measure of compliance), and hosting company type were associated with non-compliance to the state requirements. Statistical analysis was conducted using RStudio version 3.6.1.

## RESULTS

### Online ENDS vendor characteristics

In total, 68 hyperlinks were collected and reviewed per our study online search protocol. After manual review, a total of 50 of these hyperlinks were classified as online ENDS vendors. Of these online ENDS vendors, 40 (80%) used some form of age verification when visiting the homepage/hyperlink for the site from a search engine result. Among the websites that used age verification, 14 vendors (35%) required entering date of birth, 10 vendors (25%) required affirmation for 18 years or older, 8 vendors (20%) required affirmation for 21 years or older, 7 vendors (17.5%) required users to choose between 18 or 21 years of age and only 1 (2.5%) vendor used a third-party verification service. Additionally, 8 websites (36%) did not report a physical address on their homepage.

Querying of associated WHOIS data found that 29 (58%) ENDS vendor IP server locations were in the United States, with 23 located in California, 2 in Virginia, 1 in Illinois, 1 in Michigan, 1 in Utah, and 1 in Texas. The remaining 21 (42%) online ENDS vendors had IP servers located in Ontario, Canada. An analysis of the domain registration for these fifty online ENDS vendors found that 41 (82%) domains were registered in the US, with 3 (6%) in Canada, 1 (2%) in Panama, and 5 (10%) having no information about registrant country or having it masked. The names and identities of the domain registrant were not available for 32 (64%) websites. Instead, these registrant names were listed as private, redacted for privacy, or hidden using private domain masking services. Only 2 (4%) domain names were registered using the same name as the online ENDS vendor, whereas 16 (32%) were registered using personal names. Among the 16 domains registered with personal names, two were registered under the same name. Data from WHOIS also revealed that GoDaddy was the most commonly used web hosting company, accounting for 36 (72%) websites.

### Simulated shopping results

**Figure 1:**
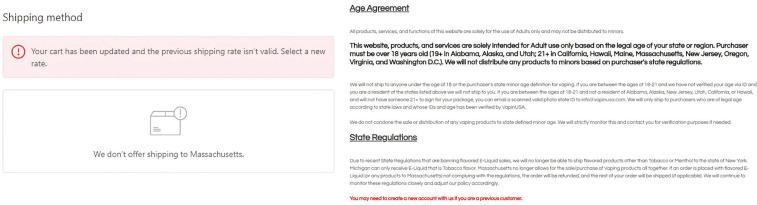
Purchase restriction on entering Massachusetts address under shipping location (Left). Age Agreement Contract Participants had to adhere to in order to advance further in the ordering process (Right)

Simulated orders were conducted on 1 October, 9 October, and 7 November for all identified online ENDS vendor websites to see if they actively offered the sale of ENDS products to Massachusetts customers, with repeated simulated orders used to determine if any changes were made for the websites’ ordering processes or selling policies. Of the 50 total online ENDS vendors reviewed, orders conducted on 1 October revealed that 38 (76%) were non-compliant, allowing the processing of simulated online purchases after entering a Massachusetts shipping address in the online order (Table 1). Of these non-compliant websites, only 10 used some form of ID verification at point-of-sale (i.e. providing proof of age or identity to finalize order). Among the 12 (24%) compliant sites that restricted sales of ENDS products to Massachusetts, restriction measures included grayed-out payment sections that prevented additional information from being entered into the online order form. Figure 1 illustrates a form of user-facing ordering constraints for online ENDS vendors with shipment restrictions.

**Table 1:** Store count by compliance, IP server location, age verification, and hosting company

*Testing date*	*Compliance*	*Number (%)*	*IP server location*		*Age verification homepage*		*Hosting company*	
			*US*	*Canada*	*Yes*	*No*	*GoDaddy.com*	*Other*
**1 October 2019**	Non-compliant	38 (76)	23	15	29	9	28	10
	Compliant	12 (24)	6	6	11	1	8	4
**9 October 2019**	Non-compliant	35 (70)	21	14	27	8	25	10
	Compliant	15 (30)	8	7	13	2	11	4
**7 November 2019**	Non-compliant	36 (72)	22	14	27	9	26	10
	Compliant	14 (28)	7	7	18	1	10	9

Breaking down compliant and non-compliant vendors by geographical location and changes in compliance rates on 1 October, 23 vendors (79.3%) with IP address located in the US and 15 (71.4%) with IP addresses located in Canada allowed simulated orders to Massachusetts. Of the 12 compliant stores that did not permit simulated orders to Massachusetts, 6 had IP server locations within the US, with the other six being located in Canada. Further simulated purchases on 9 October 2019 found that a total of 15 stores were compliant, which included three stores that changed their processes to restrict shipments to Massachusetts addresses, with two of these having IP addresses from the United States and a third with an IP address from Canada. The final set of simulated purchases was conducted on 7 November 2019 and found that a total of 14 stores (7 in the US and 7 in Canada) restricted shipments to a Massachusetts address, including one store that returned to a non-compliant status.

The association between website compliance and having an IP server location in the US was not statistically significant (p=0.738), based on compliance rates from 1 October. The lack of association persisted for subsequent simulated purchase dates. Furthermore, there was no significant association between website compliance and age verification requirement or hosting company type on any simulated purchase dates. As with testing for shipment restrictions, the differences in compliance proportions were not significantly different between US and Canada sites for any simulated purchases. Among the non-compliant websites, identity verification requirement (at point-of-sale) was found for 7 websites (30.4%) with IP addresses located in the US and 3 websites (20%) with IP addresses located in Canada on 1 October (Table 2). However, the association between IP server location and identity verification requirement among these non-compliant websites was not statistically significant (p=0.709) and these results were also consistent for subsequent simulated purchases. Since the testing window was limited, the resulting low sample size of websites yielded no significant associations between store compliance and any of the characteristics examined.

**Table 2:** ID Verification requirement and IP server location of non-compliant stores (US and Canada)

*Date*	*ID verification*	*Noncompliant stores n (%)*	*IP server location*	
			*US*	*Canada*
**1 October 2019**	Required	10 (26.3)	7	3
	Not Required	28 (73.7)	16	12
**9 October 2019**	Required	7 (20.0)	4	3
	Not Required	28 (80.0)	17	11
**7 November 2019**	Required	10 (27.8)	7	3
	Not Required	26 (72.2)	15	11

Finally, none of the 50 websites reviewed in this study sold ENDS products or other merchandise that contained THC. The only mention of THC was on labels that read ‘THC Free’ for an e-liquid or a brand named THC. This was assessed by using the internal site search function of an online ENDS vendor website to identify possible selling of THC-related products.

## DISCUSSION

Internet searches and simulated orders conducted in this study provide new insights into the compliance status of online ENDS vendors in the context of a comprehensive statewide ENDS sales ban that was enacted by Executive Order in Massachusetts for a period of approximately 3 months. Generally, age verification results align with other studies that either detected no age verification process on websites, using strategies that cannot effectively verify age, and only one that used a form of online age verification services^19,22,24^. Findings also augment prior policy and compliance studies that have identified state legislation banning direct-to-consumer shipments of internet/mail-order cigarette sales, instituting minimum age requirements, establishing vendor licensure requirements, requiring product safety requirements on online vaping/e-liquid product sales, implementing tax collection/remittances, and creating penalties and enforcement mechanisms (including against shippers, purchasers and vendors)^25-27^. Currently, there is no federal law banning online sales of tobacco or ENDS, and additional research is needed to assess what policy options are most effective in curbing online tobacco/ENDS uptake. This includes conducting empirical evaluation of implementation and effectiveness of formal legislation, executive orders, litigation, actions by regulators, and voluntary actions by the commercial sector to address online sales^25-27^.

Our study aligns with results from prior studies characterizing non-compliant characteristics of online ENDS vendors, particularly in the context of failure to adhere to state licensure or sales ban requirements, age verification, and presence of international websites^18,20,24^. At the end of our study period, a majority (n=36; 72%) of websites reviewed remained non-compliant to the Massachusetts sales ban, which expired on 11 December and was replaced with passage of legislation (An Act Modernizing Tobacco Control) of a narrower statewide retail sales ban on all flavored tobacco and ENDS products, including menthol cigarettes and flavored chewing tobacco. We detected slight increases in rates of compliance over the purchase testing phases for vendors with IP addresses in the US, thereby suggesting that a longer study period may have produced additional information about the possible association between the time and rates of compliance to assess proper implementation of the ENDS sales ban. In contrast, non-compliant sites remained open for business and ‘user-friendly’, in some cases permitting easy access to underage buyers of banned products who could simply place items in the online shopping cart and use questionable age and identity verification processes (if any) to complete the purchase.

Although ENDS products were not actually purchased in our study due to legal considerations, results provide important insights into potential challenges associated with online ENDS industry compliance with state-based tobacco sales bans, particularly in the context of ensuring appropriate identification of non-compliant sites and addressing the presence of non-domestic sellers that may import banned products into the US. Other studies have also highlighted challenges in policy implementation, including lack of legal compliance mechanisms and enforcement activities, which represent a significant challenge for future ENDS product sales bans, including the current Massachusetts ban for flavored tobacco and ENDS products^25^. For example, in the case of the Massachusetts executive order, other than a press release issued by the governor’s office, it is unclear if specific attempts were made to disseminate information about the temporary ban to online stores, particularly those not located in the state. Whether enforcement actions, penalties, or lawsuits brought against online vendors (including a December 2019 lawsuit brought by the Massachusetts Attorney General against eight ENDS companies illegal selling and delivering flavored ENDS products) will have an impact on rates of compliance remains understudied^25,28^. Further, challenges associated with online anonymity or location of business operations may make it difficult to actually identify a vendor. For example, many sites reviewed did not report their physical address and some had their website registration data (i.e. WHOIS information) removed or masked.

Internet surveillance approaches, such as that used in this study, nevertheless have the potential to identify and characterize non-compliant sites and may help with subsequent regulatory and enforcement efforts. For example, many of the online ENDS vendors detected hosted their websites in the US and on major hosting platforms (e.g. Godaddy.com), which includes terms and conditions that web stores must be ‘appropriately licensed and compliant with all local laws for the jurisdictions in which they do business’^29^. Hence, violation of state law could trigger a violation of Internet Service Provider terms that could lead to suspension or removal of a website, but such action is platform specific. However, sales bans should also be viewed as just one of the many policy mechanisms available in either the federal or state government tobacco control regulatory science toolkit, which includes restrictions on manufacturing, importing, packaging, labeling, advertising, promotion, sale, distribution, and enforcing age requirements, but also requires contextualization to the unique challenges of the online environment^30,31^.

Finally, due to limitations in the power of our sample, we were unable to identify if any statistically significant associations were present between online ENDS vendors that were compliant with the sales ban and compliance characteristics of interest (e.g. age verification, location, hosting company, etc.) Also, the possibility of no association or confounding due to other characteristics cannot be ruled out. We also failed to detect active selling of THC ENDS products in our targeted online ENDS vendor web surveillance, indicating that sites reviewed do not appear to be sources of products highly associated with EVALI-related health risks, though other non-THC nicotine products have also been associated with EVALI cases^32^.

### Limitations

Due to the short time duration of the study and the Massachusetts sales ban, sample sizes were small, thereby limiting our ability to find statistically significant differences in vendor characteristics based on compliance or non-compliance. Since it was illegal to ship ENDS products to a Massachusetts address during the study period, we could only evaluate how far the online purchasing process would progress in a simulated fashion. Specifically, conducting ‘secret shopper’ test-buys of products and shipping them to a Massachusetts address would be illegal and requires a state waiver or other exemption to conduct research on the topic, which was deemed impractical given the short duration of the sales ban itself. We also chose ENDS starter kits for simulated purchases as they were the most widespread product available and for consistency purposes, though popular cartridge-based ENDS devices could have also been used to assess website compliance and should be considered in future studies. For websites that did not ask for age verification, there were limited ways to verify if age verification was actually queried by a vendor after payment information was entered. Finally, we did not specifically assess the implementation of dissemination of information associated with the executive order, which is critical to understanding why vendors may have complied or not complied with the sales ban. Future studies should incorporate approaches to analyze information on the dissemination process of policy implementation among the general public and for online ENDS vendors. Another potential limitation of the study was a legal challenge by the vaping industry requesting an injunction to stop the ban from taking into effect. Though the State Supreme Court declined to halt the ban, it was reissued on 28 October as an emergency regulation which would expire on 24 December, a month earlier than the initial ban’s intended expiration date. Uncertainty regarding the legal status of the sales ban due to these challenges may have impacted vendor compliance but was not measured in this study.

## CONCLUSIONS

The Massachusetts executive order allowed for an investigation into vendor compliance during a temporary and comprehensive sales ban of ENDS products that also included online sales. Our study also confirmed other concerning characteristics detected in other studies, including lack of age verification and presence of international sellers. However, future research is critically needed to explore how sales ban policy implementation impacts compliance of the virtual tobacco and ENDS marketplace, including for numerous partial sales bans now at the federal, state, and local levels, including the FDA’s new guidelines outlining enforcement actions on the sale of any flavored, cartridge-based ENDS product (excluding menthol flavored), sold both offline and online. Complementing policy implementation research should also be active online monitoring to further characterize non-compliant online ENDS vendors for purposes of identifying violating sites, issuing penalties, effectuating website suspensions, and removing prohibited products in order to ensure that the internet does not become the future safe haven for prohibited ENDS access.
